# Supplementary figures and images for: Rab10 inactivation promotes AMPAR trafficking and spine enlargement during long-term potentiation
Source: eLife. 2025 Sep 23;13:RP103879. doi: 10.7554/eLife.103879 (PMC12456950; doi:10.7554/eLife.103879)

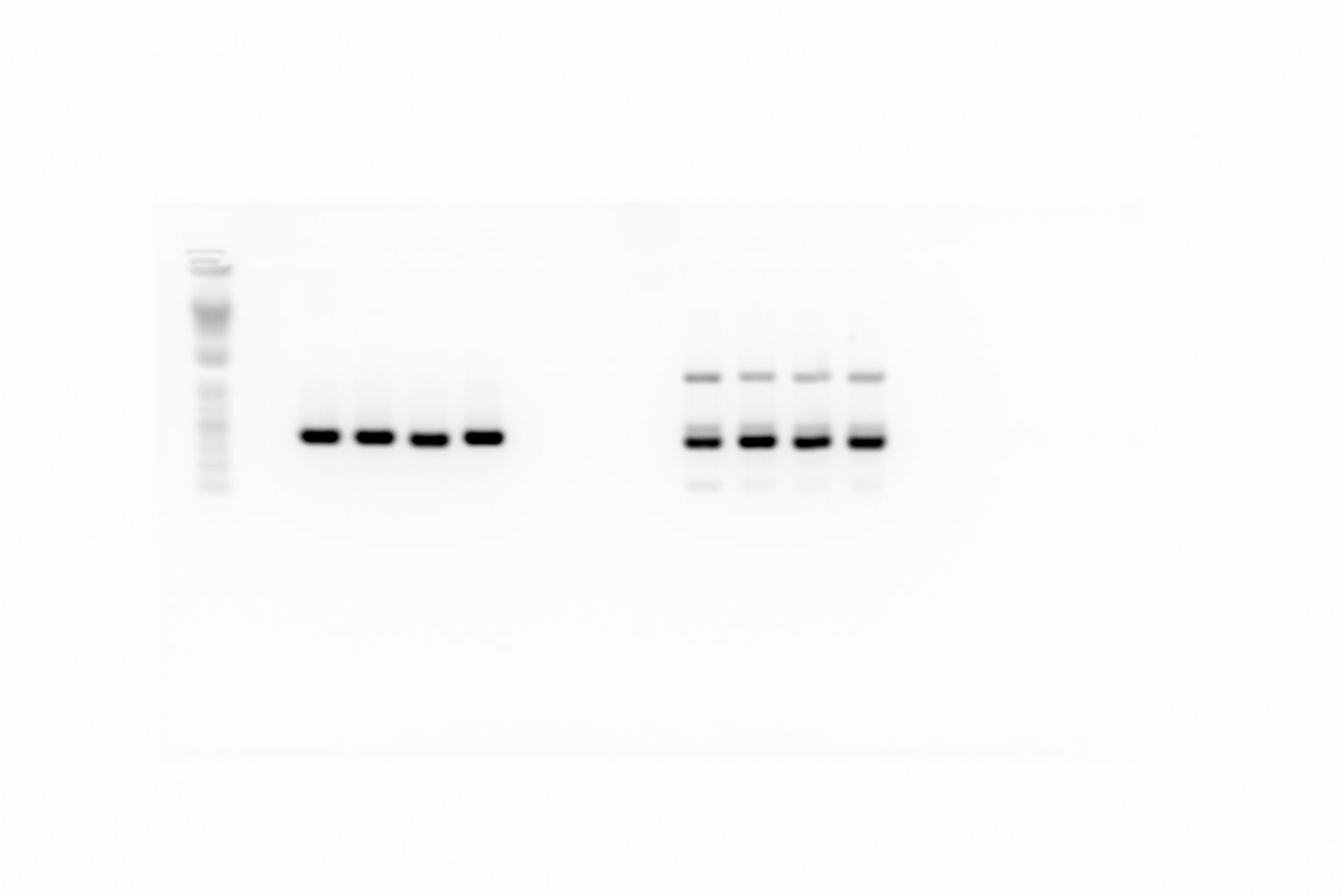

Supplement: Figure 1—figure supplement 1—source data 1. [file elife-103879-fig1-figsupp1-data1.zip › Figure 1 - figure supplement 1G Bottom Panel original.jpg]

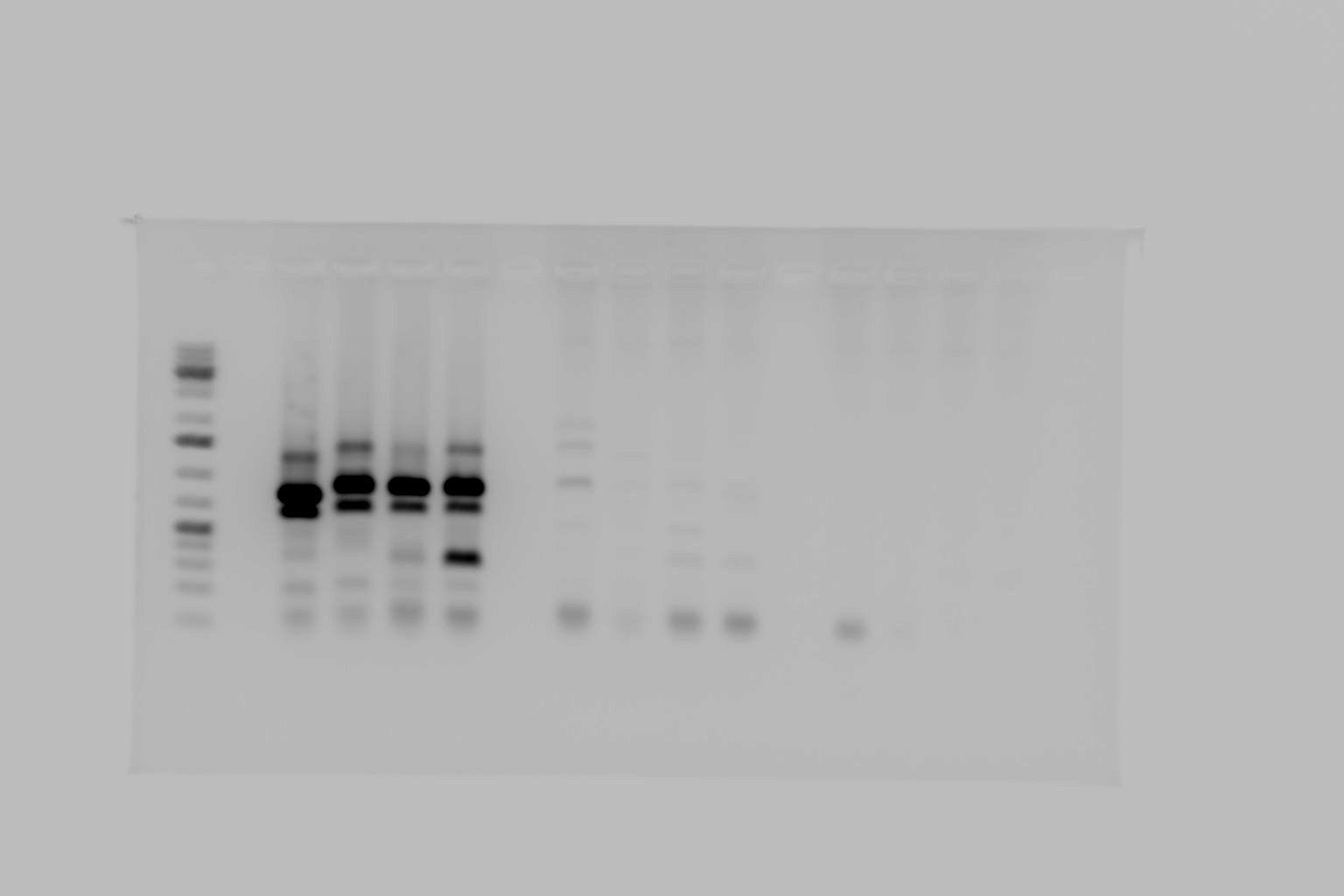

Supplement: Figure 1—figure supplement 1—source data 1. [file elife-103879-fig1-figsupp1-data1.zip › Figure 1 - figure supplement 1G Top Panel original.jpg]

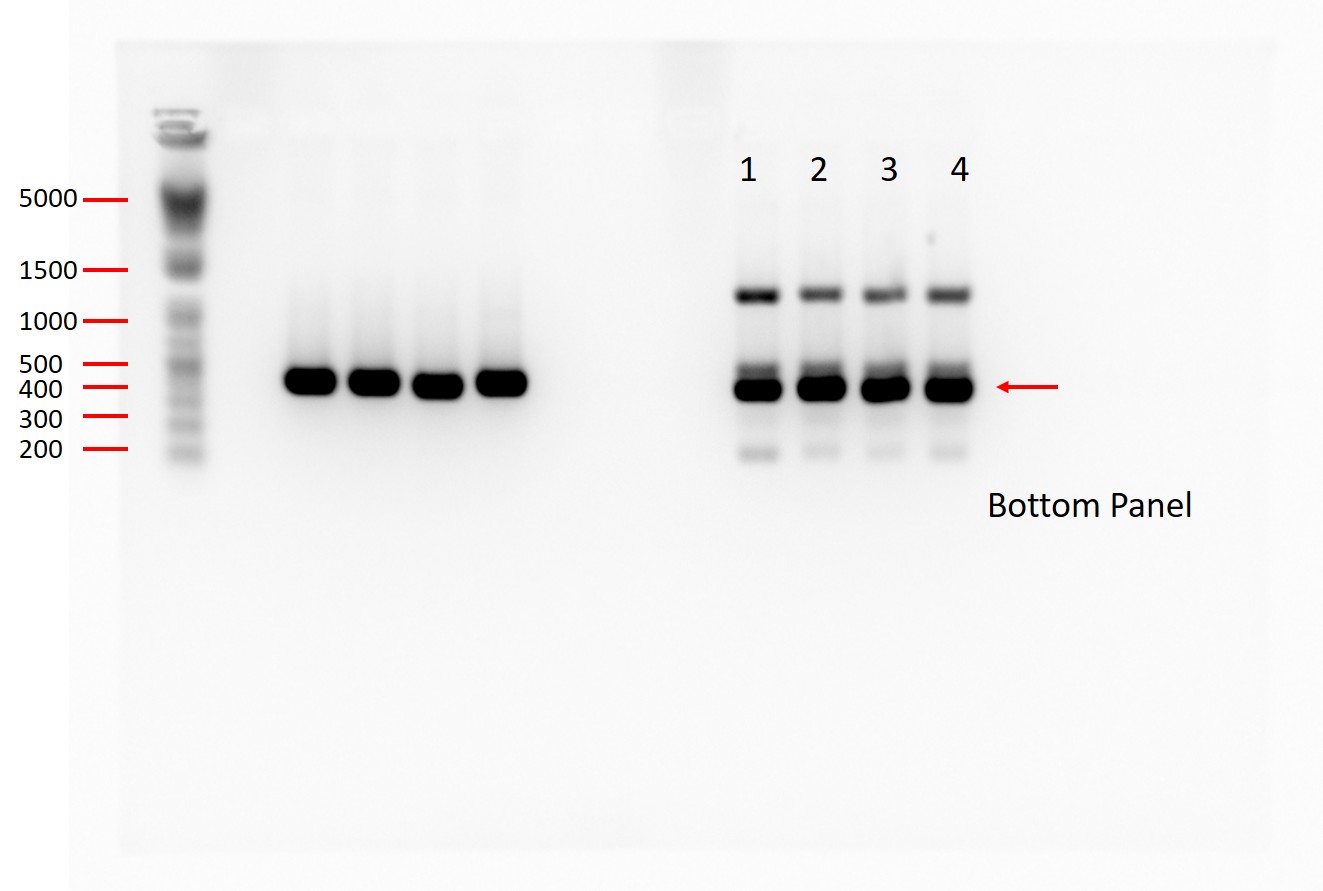

Supplement: Figure 1—figure supplement 1—source data 2. [file elife-103879-fig1-figsupp1-data2.zip › Figure 1 - figure supplement 1G Bottom Panel with annotation.jpg]

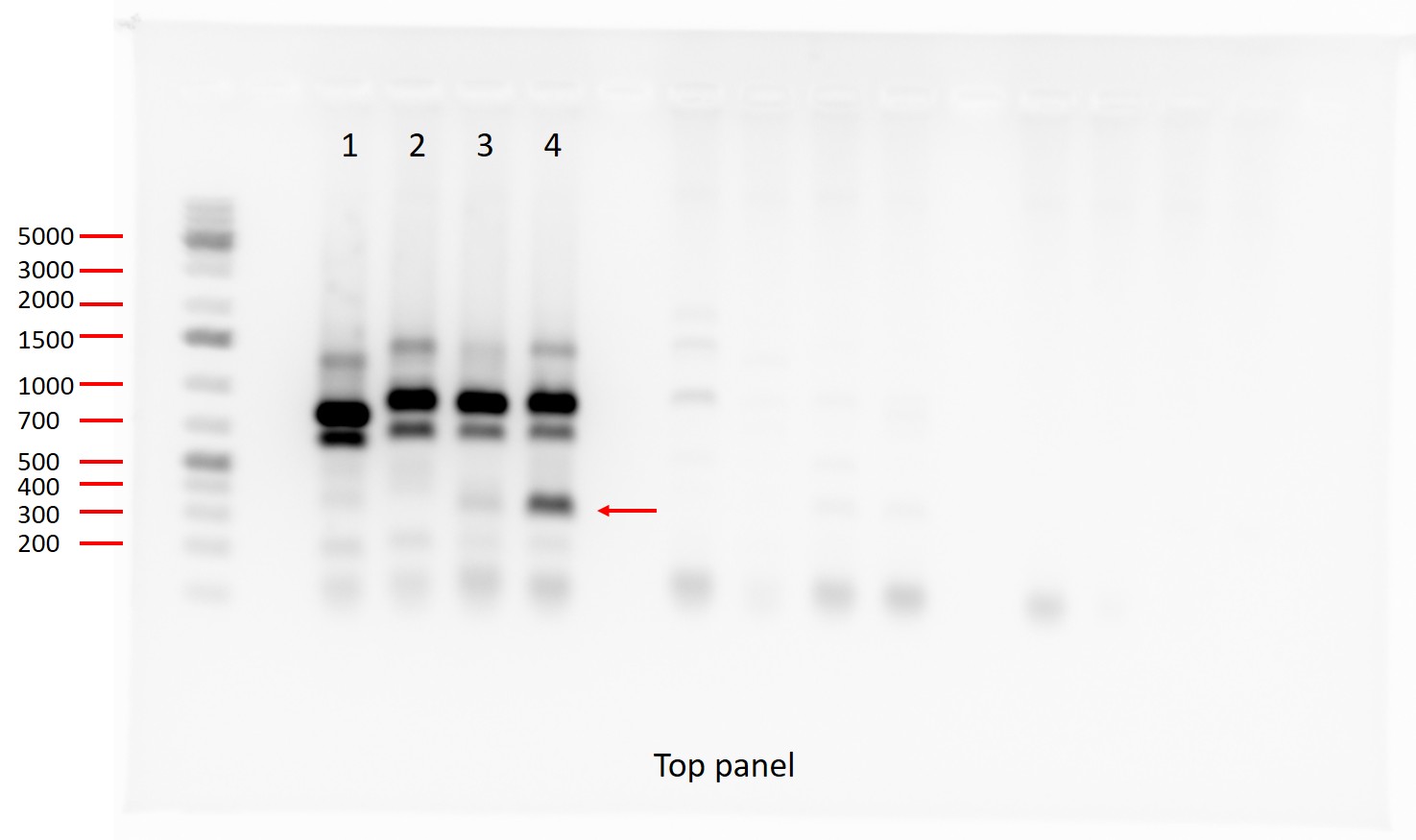

Supplement: Figure 1—figure supplement 1—source data 2. [file elife-103879-fig1-figsupp1-data2.zip › Figure 1 - figure supplement 1G Top Panel with annotation.jpg]

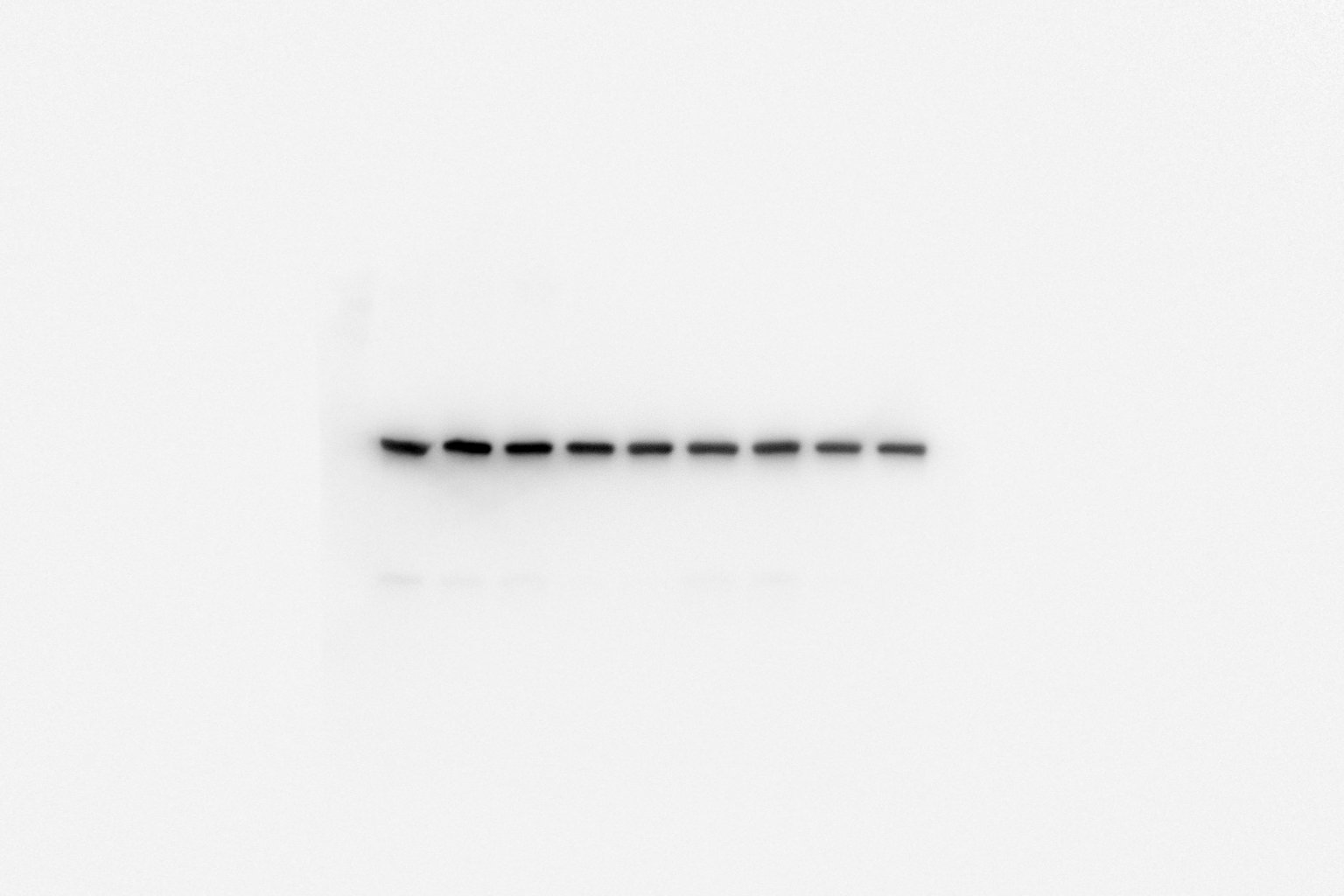

Supplement: Figure 4—figure supplement 1—source data 1. [file elife-103879-fig4-figsupp1-data1.zip › Figure 4 - figure supplement 1E bottom panel original.jpg]

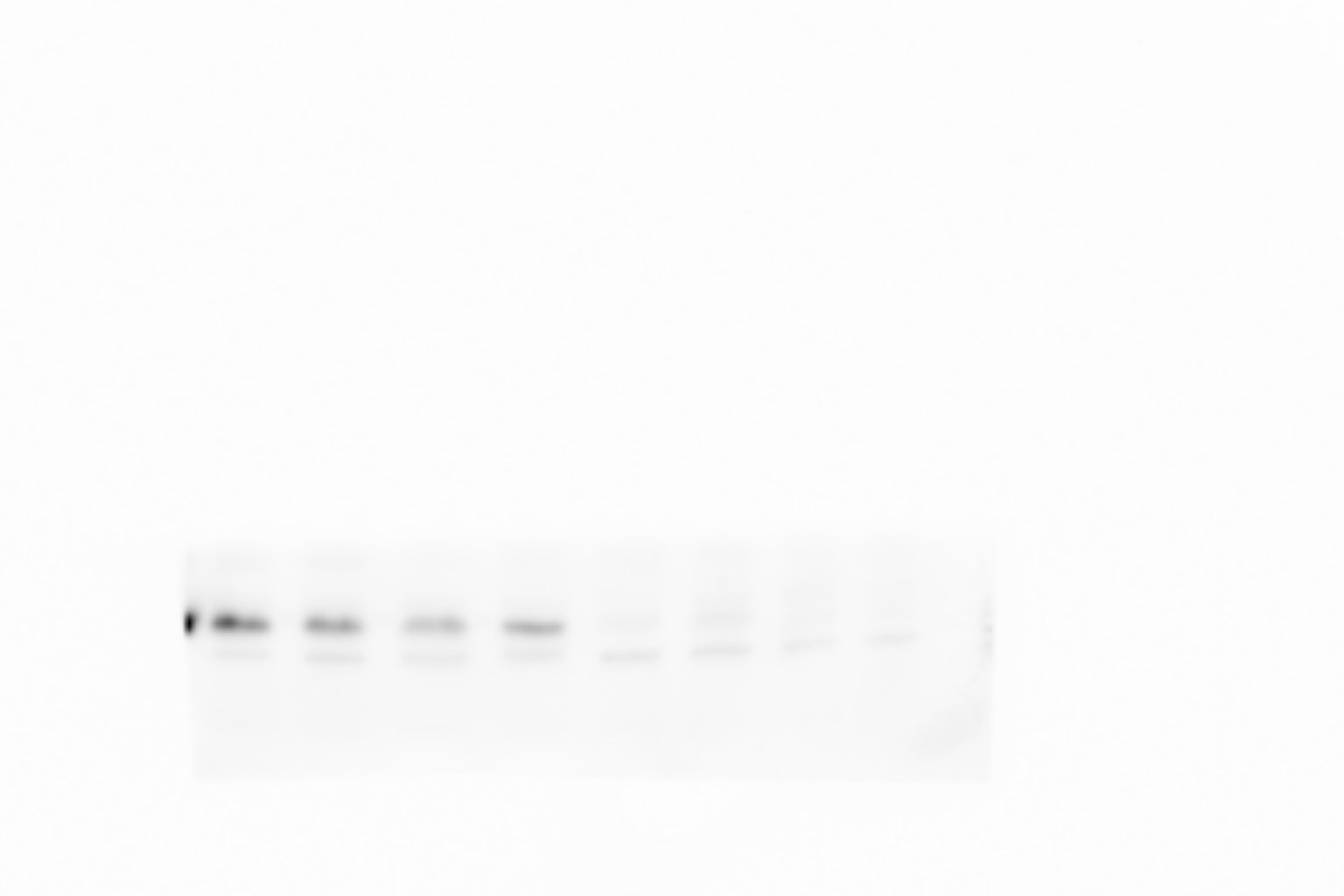

Supplement: Figure 4—figure supplement 1—source data 1. [file elife-103879-fig4-figsupp1-data1.zip › Figure 4 - figure supplement 1E middle panel original.jpg]

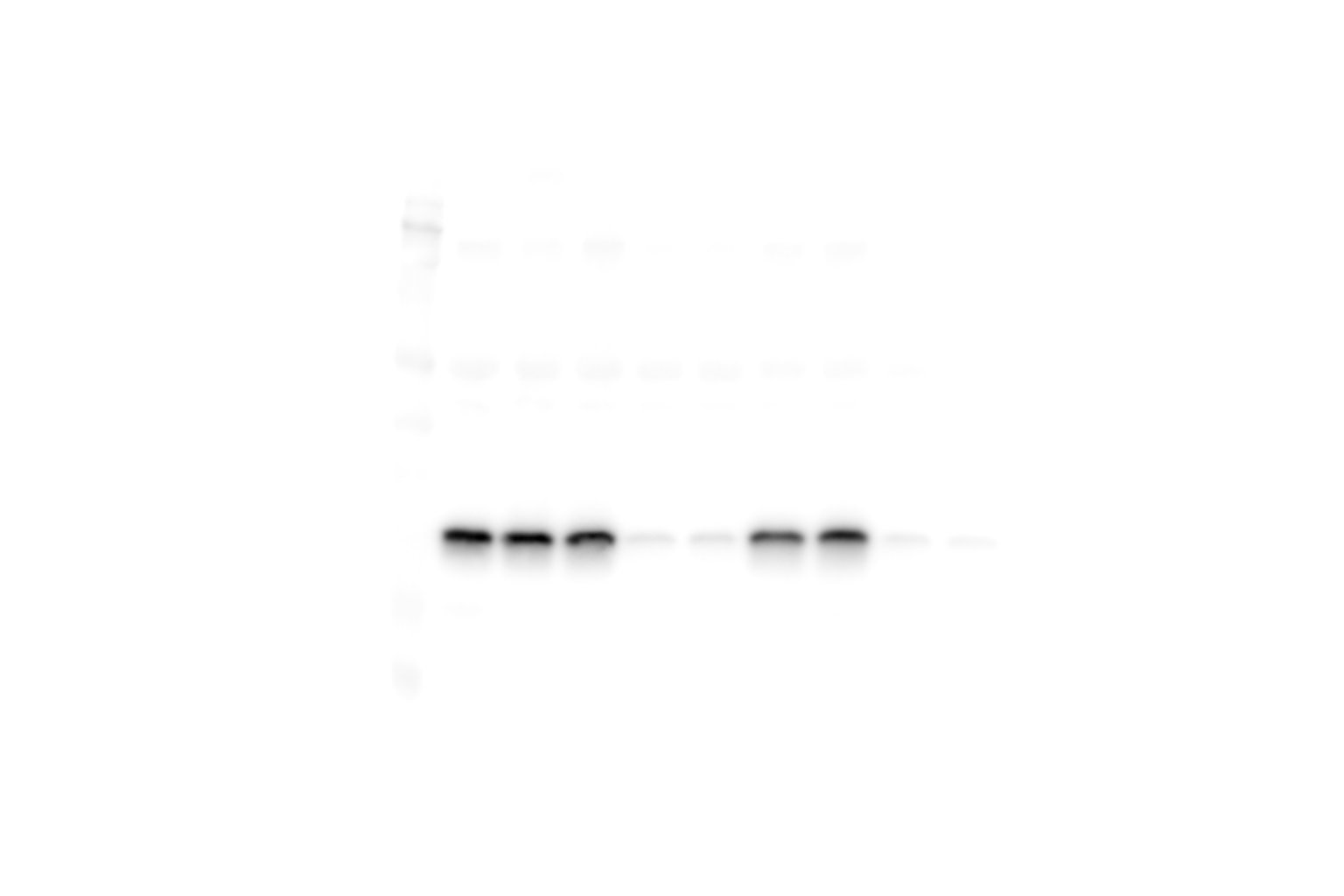

Supplement: Figure 4—figure supplement 1—source data 1. [file elife-103879-fig4-figsupp1-data1.zip › Figure 4 - figure supplement 1E top panel original.jpg]

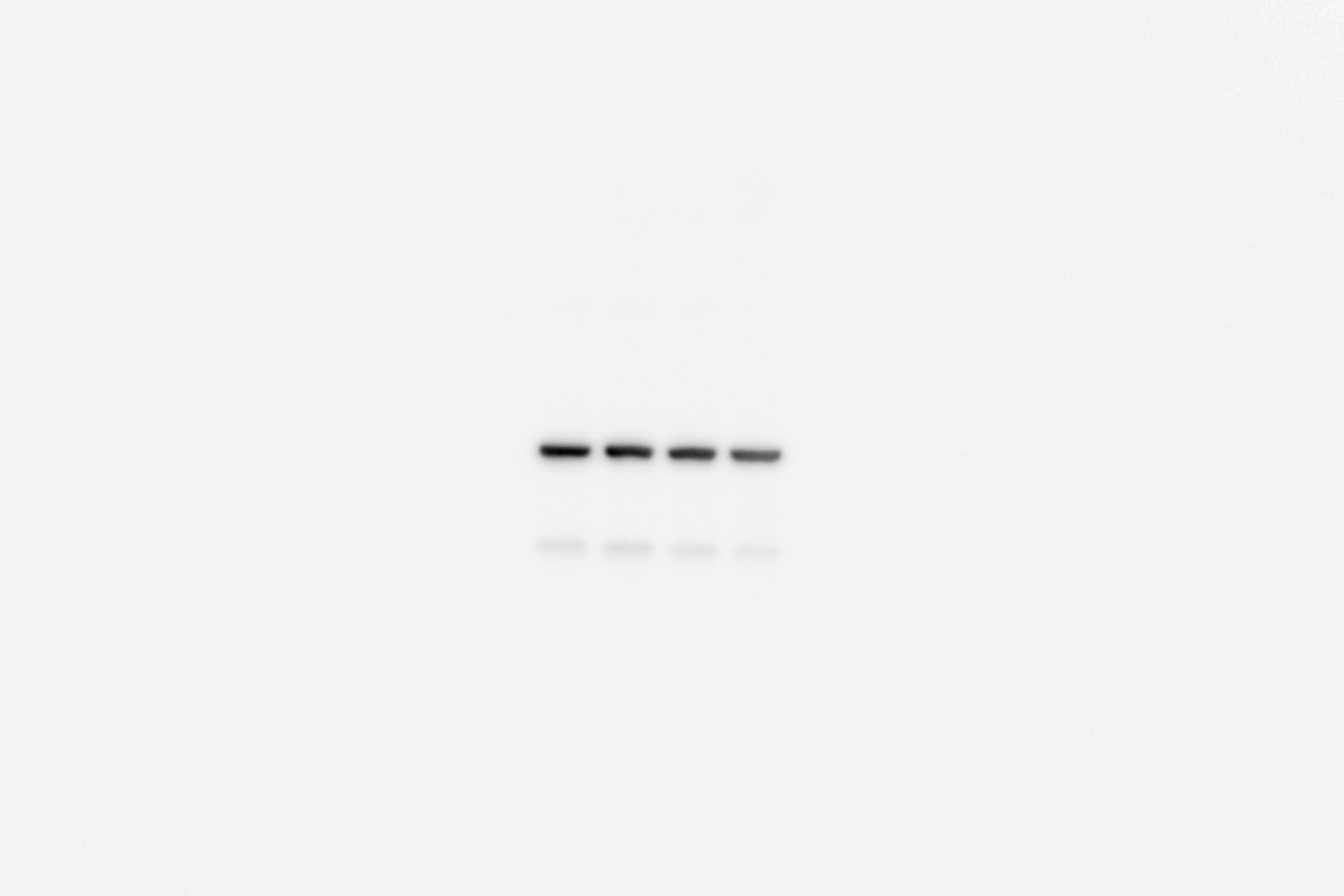

Supplement: Figure 4—figure supplement 1—source data 1. [file elife-103879-fig4-figsupp1-data1.zip › Figure 4 - figure supplement 1F bottom panel original.jpg]

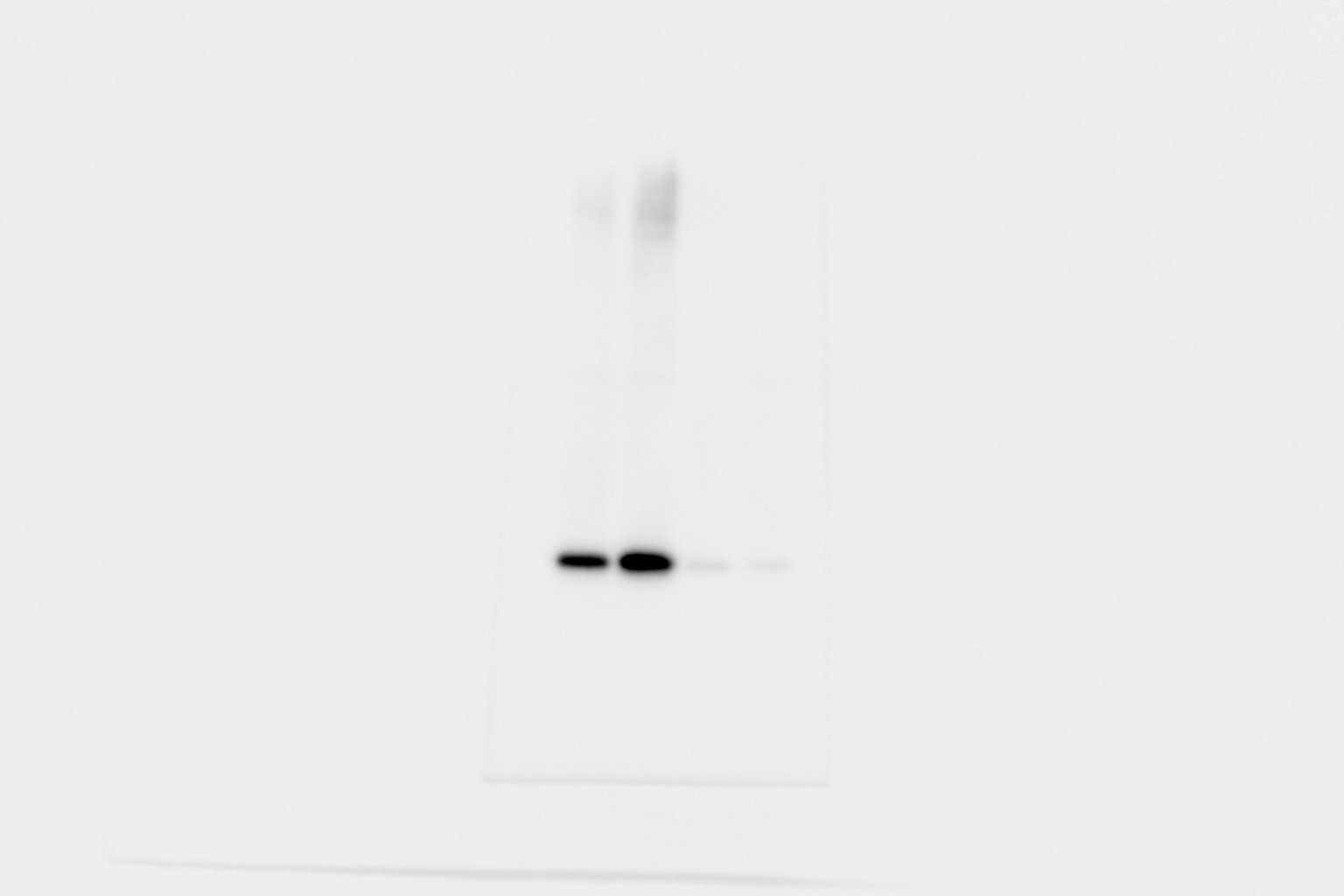

Supplement: Figure 4—figure supplement 1—source data 1. [file elife-103879-fig4-figsupp1-data1.zip › Figure 4 - figure supplement 1F top panel original.jpg]

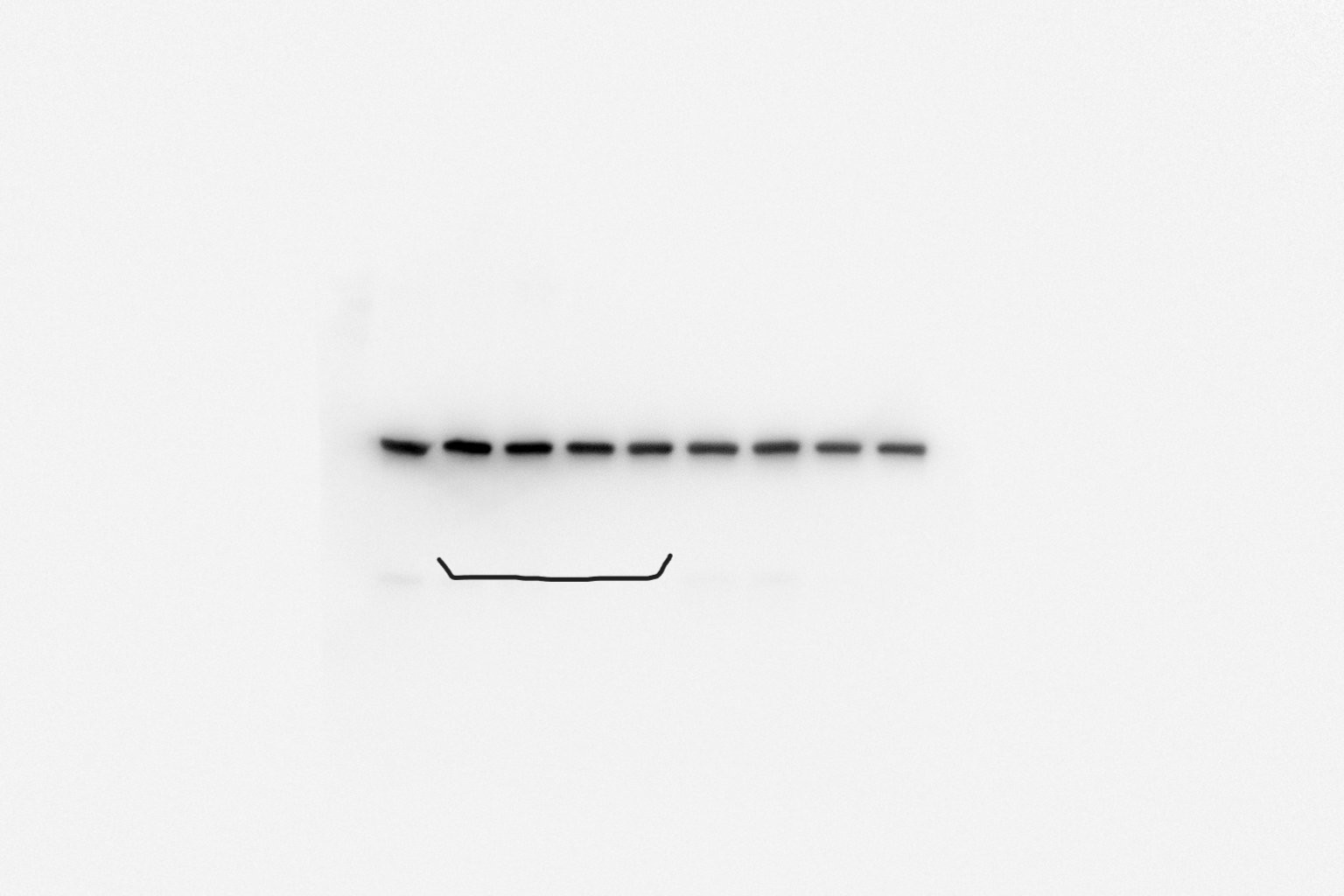

Supplement: Figure 4—figure supplement 1—source data 2. [file elife-103879-fig4-figsupp1-data2.zip › Figure 4 - figure supplement 1E bottom panel with annotation.jpg]

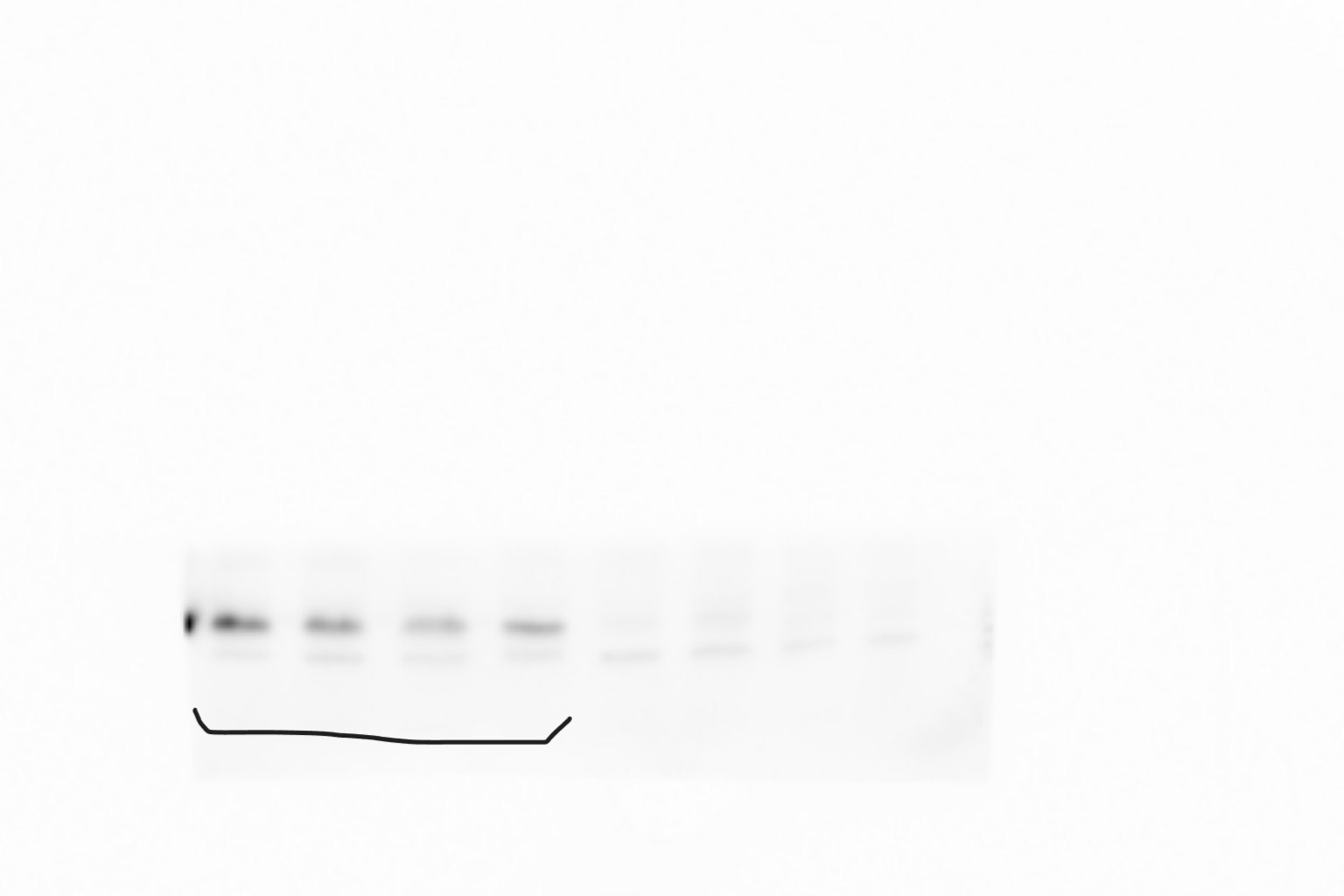

Supplement: Figure 4—figure supplement 1—source data 2. [file elife-103879-fig4-figsupp1-data2.zip › Figure 4 - figure supplement 1E middle panel with annotation.jpg]

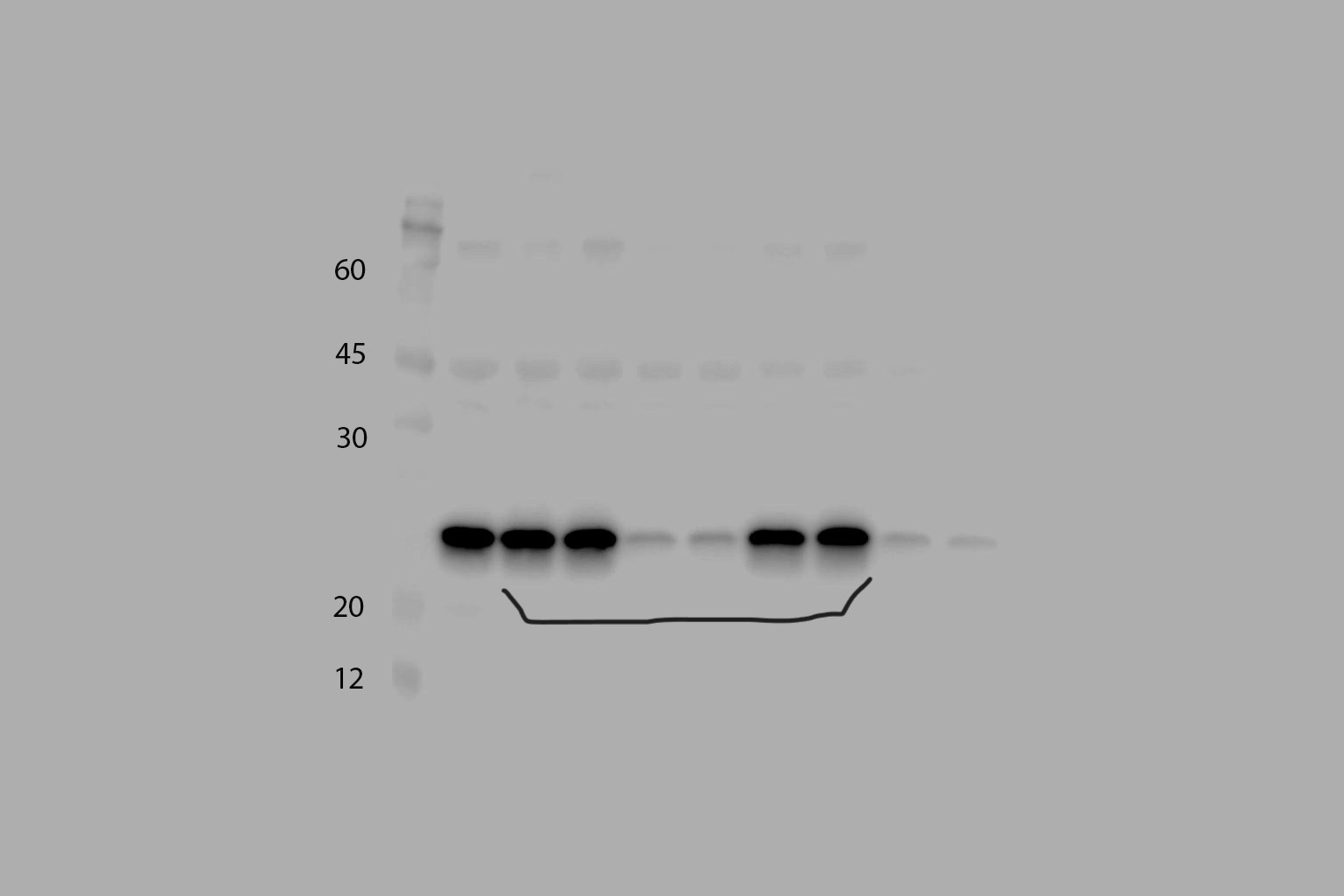

Supplement: Figure 4—figure supplement 1—source data 2. [file elife-103879-fig4-figsupp1-data2.zip › Figure 4 - figure supplement 1E top panel with annotation.jpg]

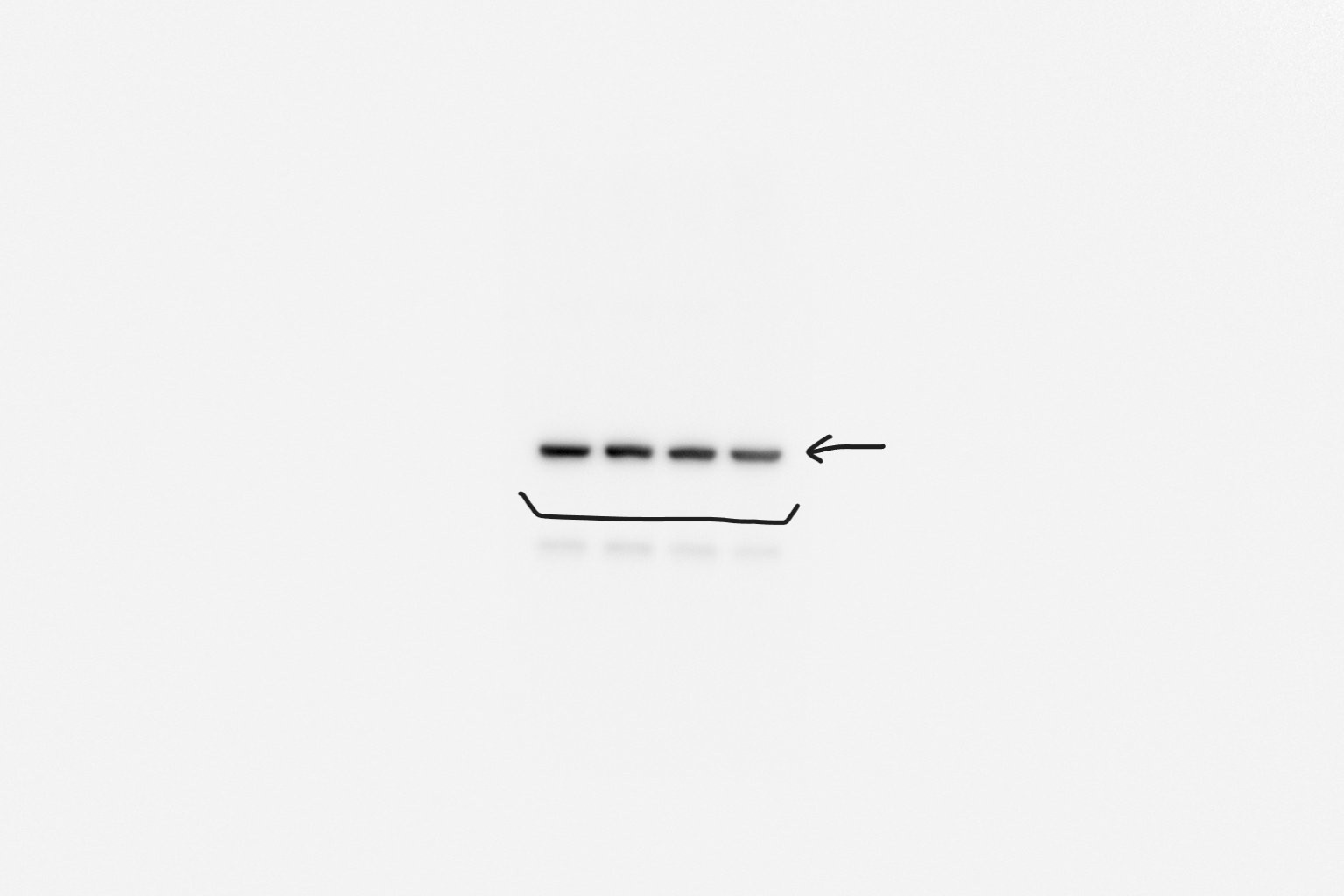

Supplement: Figure 4—figure supplement 1—source data 2. [file elife-103879-fig4-figsupp1-data2.zip › Figure 4 - figure supplement 1F bottom panel with annotation.jpg]

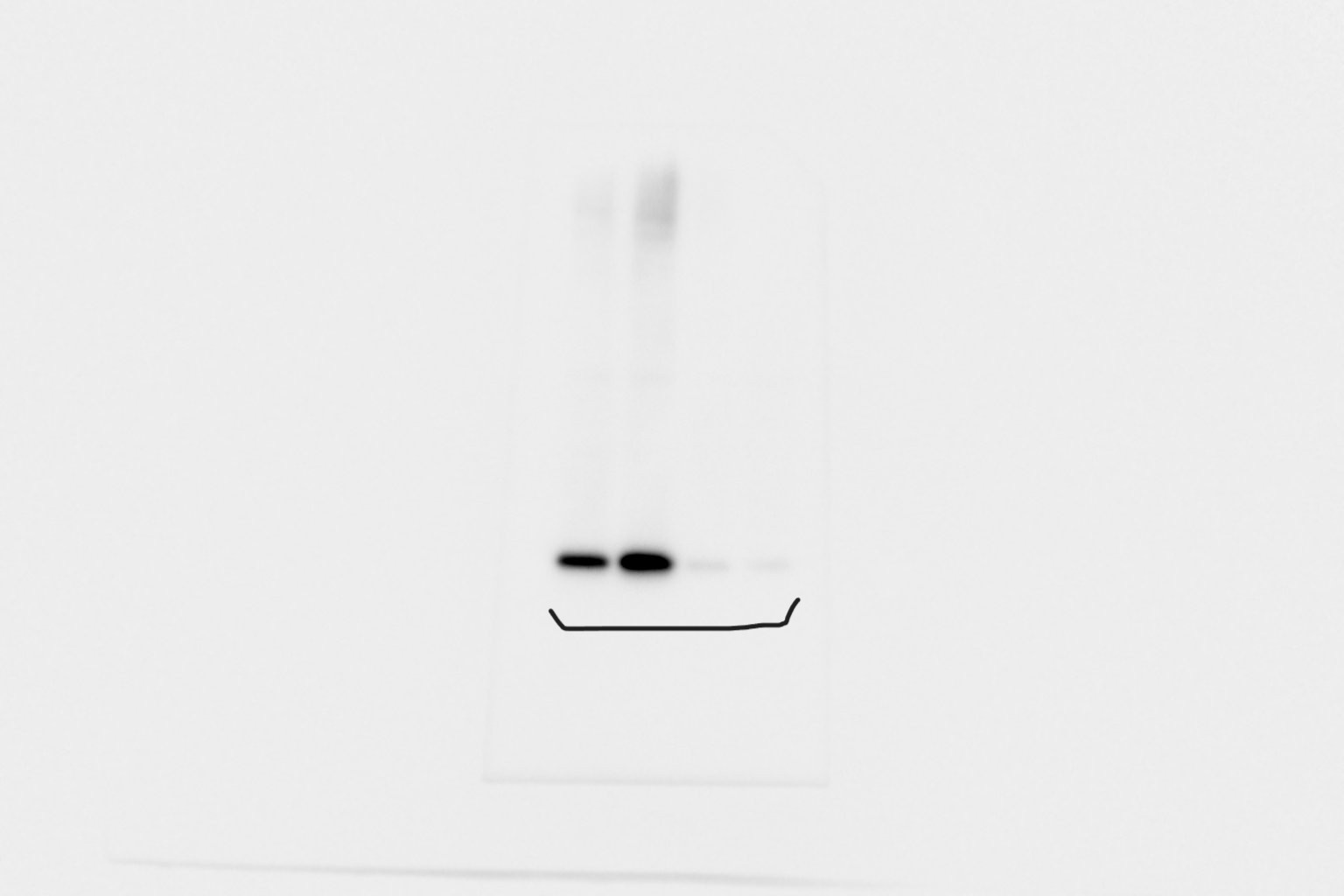

Supplement: Figure 4—figure supplement 1—source data 2. [file elife-103879-fig4-figsupp1-data2.zip › Figure 4 - figure supplement 1F top panel with annotation.jpg]
